# Supplementary material for: A hierarchical Bayesian model to find brain-behaviour associations in incomplete data sets
Source: Neuroimage. 2022 Apr 1;249:118854. doi: 10.1016/j.neuroimage.2021.118854 (PMC8861855; doi:10.1016/j.neuroimage.2021.118854)
Supplement: Supplementary Data S1 — Supplementary Raw Research Data. This is open data under the CC BY license http://creativecommons.org/licenses/by/4.0/ [file mmc1.pdf]

Supplementary Material: A hierarchical Bayesian model to find brain-behaviour associations in incomplete data sets

Fabio S. Ferreira[a,b,*], Agoston Mihalik[a,b], Rick A. Adams[a,b,c], John Ashburner[c,**], Janaina Mourao-Miranda[a,b,**]

[a] *Centre for Medical Image Computing, Department of Computer Science, University College London, London, UK*
[b] *Max Planck University College London Centre for Computational Psychiatry and Ageing Research, University College London, UK*
[c] *Wellcome Centre for Human Neuroimaging, University College London, London, UK*

Keywords: Multivariate methods, group factor analysis, Bayesian inference, missing data, brain connectivity, behaviour

1. Materials and Methods

1.1. Additional GFA experiments on synthetic data

We ran GFA experiments on the following selections of synthetic data:

1. *Complete data* (all models were initialised with $K = 30$):
   (a) low dimensional data ($D_1 = 50$ and $D_2 = 30$) was generated using the same parameters described in Section 2.4.1 of the main text.
   (b) high dimensional data ($D_1 = 20000$ and $D_2 = 200$) was generated using the same parameters described in Section 2.4.1 of the main text.
2. *Incomplete data* (all models were initialised with $K = 15$):
   (a) the elements of $\mathbf{X}^{(2)}$ deviating more than 1σ (i.e., standard deviation) from the mean (i.e, $x_{dn} > \mu + \sigma$ and $x_{dn} < \mu - \sigma$) were removed from the synthetic data generated in the supplementary experiment 1a, which led to approximately 30% of missing values in $\mathbf{X}^{(2)}$.
   (b) 10% of the rows of $\mathbf{X}^{(1)}$ and 20% of the elements of $\mathbf{X}^{(2)}$ were randomly removed from the low dimensional data generated in the supplementary experiment 1a.
   (c) 10% of the rows of $\mathbf{X}^{(1)}$ and 20% of the elements of $\mathbf{X}^{(2)}$ were randomly removed from the high dimensional data generated in the supplementary experiment 1b.

*Corresponding author. Centre for Medical Image Computing (CMIC), 90 High Holborn, Holborn, London WC1V 6LJ.
**These authors contributed equally to this work.
Email address: `fabio.ferreira.16@ucl.ac.uk` (Fabio S. Ferreira)

1.2. CCA experiments on synthetic data

In order to assess the CCA performance in complete and incomplete data sets, we generated data using the parameters described in Section 2.4.1 of the main text and ran experiments on the following selections of the data:

- *Complete data*
- *Incomplete data*
  - 20% of the elements of $\mathbf{X}^{(1)}$ and 40% of the elements of $\mathbf{X}^{(2)}$ were randomly removed.
  - the elements of $\mathbf{X}^{(1)}$ and $\mathbf{X}^{(2)}$ deviating more than 1σ from the mean were removed, which led to approximately 30% of missing values in each data modality.

The missing values were imputed using the median. The statistical significance of the CCA modes was estimated by permutation inference, in which the rows of $\mathbf{X}^{(2)}$ were permuted 1000 times and CCA was run after each permutation. For each CCA mode, we compute a p-value to assess whether the "true" canonical correlation (i.e., the canonical correlation of the respective CCA mode obtained without permuting the data) was larger than the null distribution of permuted canonical correlations of the first CCA mode (equivalent to a maximum statistics approach). To obtain an equivalent representation of a single latent variable for CCA (comparable to a latent factor in GFA), the canonical scores $\mathbf{U}^T\mathbf{X}^{(1)}$ and $\mathbf{V}^T\mathbf{X}^{(2)}$, where $\mathbf{U} \in \mathbb{R}^{D_1 \times K}$ and $\mathbf{V} \in \mathbb{R}^{D_2 \times K}$, were averaged.

These experiments were also run using our GFA extension without imputing the missing values. The performance of the models were assessed by visually comparing the inferred latent factors. The incomplete data experiments were different from those described in Section 2.4.1 of the main text because we wanted to show the potential of GFA to handle missing data when more than one modality had missing values. Moreover, it would be of little interest to run CCA with missing rows because, in practice, one would not impute the missing values but rather remove the rows in both data modalities.

1.3. CCA experiments on the HCP data

To compare our GFA results with CCA, we applied a CCA analysis similar to the one proposed by Smith et al. (2015) to the HCP data used in the GFA experiments. In summary, we reduced the dimensionality of both data modalities using Principal Component Analysis (fixing the number of principal components in each data modality to 100) and applied CCA to these reduced data matrices. The statistical significance of the CCA modes was estimated by permutation inference, in which the subjects of the non-imaging matrix were permuted 10,000 times respecting the family structure of the data (Winkler et al., 2015). The permutation approach was identical to that described in supplementary Section 1.2. For more details of the analysis, see Smith et al. (2015).

1.4. Surface plots

The surface plots illustrate maps of brain connection strength increases/decreases, which were obtained by weighting each node's parcel map with the GFA/CCA edge-strengths (the loadings were multiplied by the sign of the population mean correlation) summed across the edges connected to the node. We used the node's parcel maps provided as a cifti file (named *melodic_IC_ftb.dlabel.nii*) in the group ICA folder (named *groupICA_3T_HCP1200_MSMAll_d200.ica*). In this file, one can find the number of the ICA component that each vertex is most likely to belong to.

2. Results

2.1. Additional GFA experiments on synthetic data

The model parameters were correctly inferred using low (Supplementary Fig. 1a) ($\hat{\tau}^{(1)} \approx 5.10$ and $\hat{\tau}^{(2)} \approx 9.98$) and high (Supplementary Fig. 1b) ($\hat{\tau}^{(1)} \approx 5.01$ and $\hat{\tau}^{(2)} \approx 9.97$) dimensional synthetic data, when the model was initialised with $K = 30$. The most relevant shared and modality-specific factors were correctly estimated in both experiments.

In supplementary experiment 2a (the elements of $\mathbf{X}^{(2)}$ deviating more than 1σ from the mean were removed), taking into account the difficulty of the task our GFA approach recovered the model parameters fairly well ($\hat{\tau}^{(1)} \approx 5.04$ and $\hat{\tau}^{(2)} \approx 11.72$), whereas the median imputation approach failed to estimate the noise parameter of the second modality ($\hat{\tau}^{(1)} \approx 5.03$ and $\hat{\tau}^{(2)} \approx 6.95$) and the third factor (i.e., the factor specific to $\mathbf{X}^{(2)}$) was erroneously identified (Supplementary Fig. 2a). Furthermore, our approach performed better in the multi-output prediction task (Supplementary Fig. 3). The proposed GFA extension predicted missing data accurately ($\rho = 0.929 \pm 0.021$).

In the supplementary experiments 2b (10% of the rows of $\mathbf{X}^{(1)}$ and 20% of the elements of $\mathbf{X}^{(2)}$ were randomly removed from low dimensional synthetic data) and 2c (10% of the rows of $\mathbf{X}^{(1)}$ and 20% of the elements of $\mathbf{X}^{(2)}$ were randomly removed from high dimensional synthetic data), our approach inferred the model parameters correctly in low ($\hat{\tau}^{(1)} \approx 5.01$ and $\hat{\tau}^{(2)} \approx 10.15$) and high dimensional ($\hat{\tau}^{(1)} \approx 5.03$ and $\hat{\tau}^{(2)} \approx 9.97$) data sets, respectively. The median imputation approach failed to estimate the model parameters in both experiments ($\hat{\tau}^{(1)} \approx 6.23$ and $\hat{\tau}^{(2)} \approx 6.39$ in supplementary experiment 2b (Supplementary Fig. 2b); $\hat{\tau}^{(1)} \approx 6.33$ and $\hat{\tau}^{(2)} \approx 3.95$ in supplementary experiment 2c (Supplementary Fig. 2c)). The performance of both approaches in the multi-output prediction task was similar and below chance level (Supplementary Fig. 3). Our GFA extension predicted reasonably well the missing observations in both modalities (supplementary experiment 2b: $\rho = 0.675 \pm 0.031$ and $\rho = 0.779 \pm 0.022$ for the missing values in $\mathbf{X}^{(1)}$ and $\mathbf{X}^{(2)}$, respectively; supplementary experiment 2c: $\rho = 0.627 \pm 0.012$ and $\rho = 0.859 \pm 0.003$ for the missing values in $\mathbf{X}^{(1)}$ and $\mathbf{X}^{(2)}$, respectively).

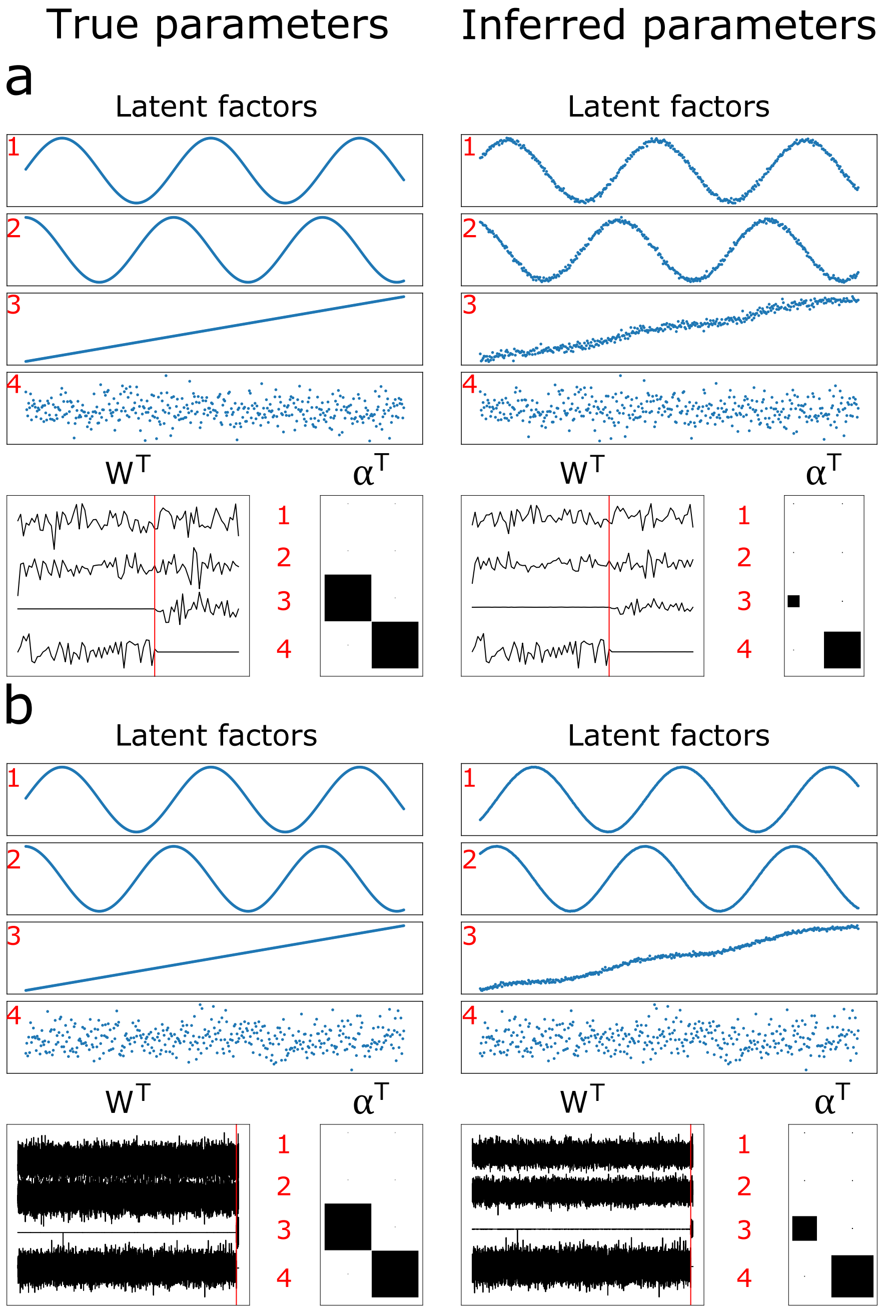

Fig. 1. True and inferred latent factors and model parameters obtained in the supplementary experiments **(a)** 1a (low dimensional complete data) and **(b)** 1b (high dimensional complete data) using $K = 30$ latent factors to initialise the models. The latent factors and parameters used to generate the data are plotted on the left-hand side, and the ones inferred are plotted on the right-hand side. The four rows on the top represent the 4 latent factors. The loading matrices of the first and second data modality are represented on the left and right-hand side of the red line in $\mathbf{W}^T$, respectively. The alphas of the first and second data modality are shown in the form of a Hinton diagram in the first and second columns of $\boldsymbol{\alpha}^T$, respectively, where the alphas are proportional to the area of the squares. The small black dots and big black squares represent active and inactive factors, respectively.

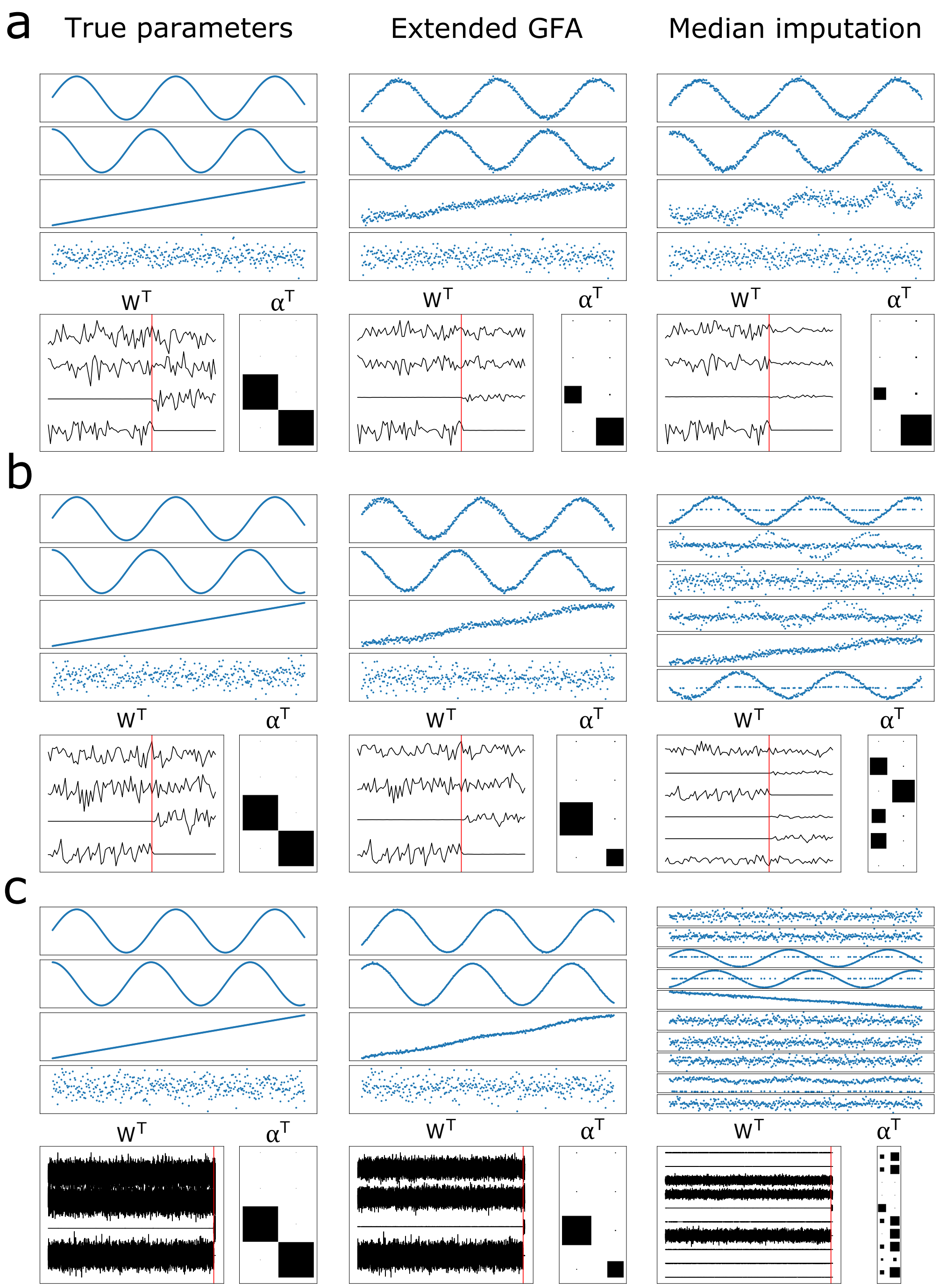

Fig. 2. True and inferred latent factors and model parameters obtained in the supplementary experiments **(a)** 2a (the elements of $\mathbf{X}^{(2)}$ deviating more than 1σ from the mean were removed), **(b)** 2b (10% of the rows of $\mathbf{X}^{(1)}$ and 20% of the elements of $\mathbf{X}^{(2)}$ were randomly removed from low dimensional synthetic data) and **(c)** 2c (10% of the rows of $\mathbf{X}^{(1)}$ and 20% of the elements of $\mathbf{X}^{(2)}$ were randomly removed from high dimensional synthetic data). **(Left column)** latent factors and model parameters used to generate the data. **(Middle column)** latent factors and parameters inferred using the proposed GFA extension. **(Right column)** latent factors and parameters inferred using the median imputation approach. The loading matrices ($\mathbf{W}^T$) and alphas ($\boldsymbol{\alpha}^T$) can be interpreted as in Supplementary Fig. 1.

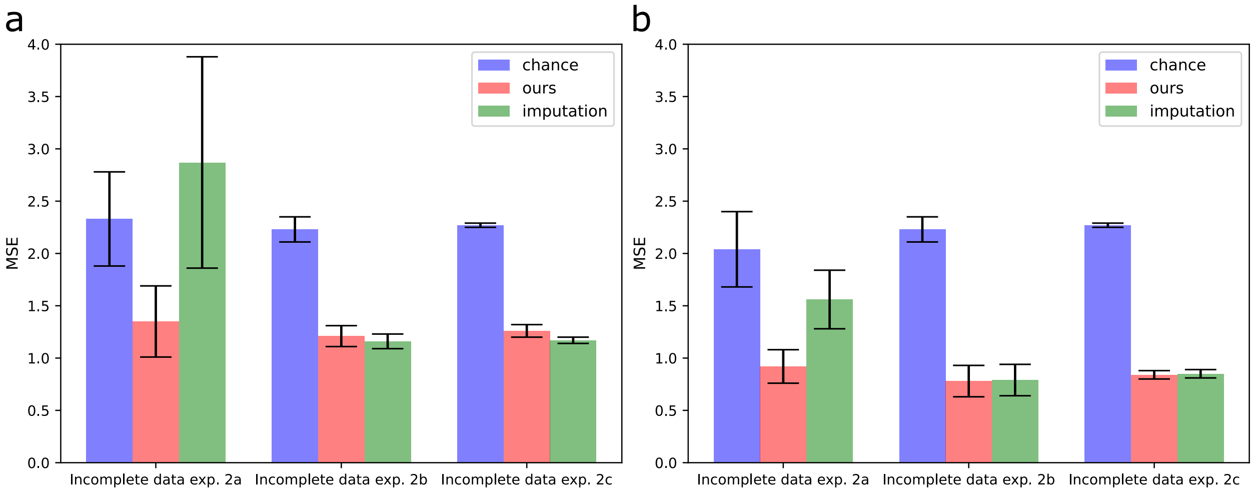

Fig. 3. Prediction errors of the multi-output prediction tasks obtained in the supplementary experiments with incomplete data. The bars and error bars correspond to the mean and standard deviation of the MSEs across 10 initialisations, respectively. **(a)** MSEs between the test observations $\mathbf{X}^{(1)^\star}$ and the mean predictions $\mathbb{E}[\mathbf{X}^{(1)^\star}|\mathbf{X}^{(2)^\star}]$ are shown for all experiments; **(b)** MSEs between $\mathbf{X}^{(2)^\star}$ and $\mathbb{E}[\mathbf{X}^{(2)^\star}|\mathbf{X}^{(1)^\star}]$ are shown for all experiments. ours - proposed GFA approach; imputation - median imputation approach; chance - chance level. Incomplete data exp. 2a - the elements of $\mathbf{X}^{(2)}$ deviating more than 1σ from the mean were removed; incomplete data exp. 2b - 10% of the rows of $\mathbf{X}^{(1)}$ and 20% of the elements of $\mathbf{X}^{(2)}$ were randomly removed from low dimensional synthetic data; incomplete data exp. 2c - 10% of the rows of $\mathbf{X}^{(1)}$ and 20% of the elements of $\mathbf{X}^{(2)}$ were randomly removed from high dimensional synthetic data.

Table 1: Most relevant shared and modality-specific factors obtained in the complete high dimensional synthetic data (supplementary experiment 1b) according to the proposed criteria. Factors explaining more than 7.5% variance within any data modality were considered most relevant. A factor was considered shared if $0.001 \leq r_k \leq 300$, specific to $\mathbf{X}^{(2)}$ if $r_k > 300$ or specific $\mathbf{X}^{(1)}$ if $r_k < 0.001$. rvar - relative variance explained; var - variance explained; r_k - ratio between the variance explained by $\mathbf{w}_k^{(2)}$ and $\mathbf{w}_k^{(1)}$.

Factors		**rvar** (%) $\mathbf{X}^{(1)}$	**rvar** (%) $\mathbf{X}^{(2)}$	**var** (%) $\mathbf{X}^{(1)}$	**var** (%) $\mathbf{X}^{(2)}$	$\mathbf{r_k}$ $\text{var}_{\mathbf{w}_k^{(2)}}/\text{var}_{\mathbf{w}_k^{(1)}}$
**Shared**	1	25.09	46.03	15.39	0.19	0.01
	2	25.47	35.44	15.62	0.15	9.6×10^{-3}
**Specific**	3	2.88×10^{-4}	18.53	1.76×10^{-4}	0.08	442.85
	4	49.44	8.80×10^{-5}	30.32	3.71×10^{-7}	1.22×10^{-8}

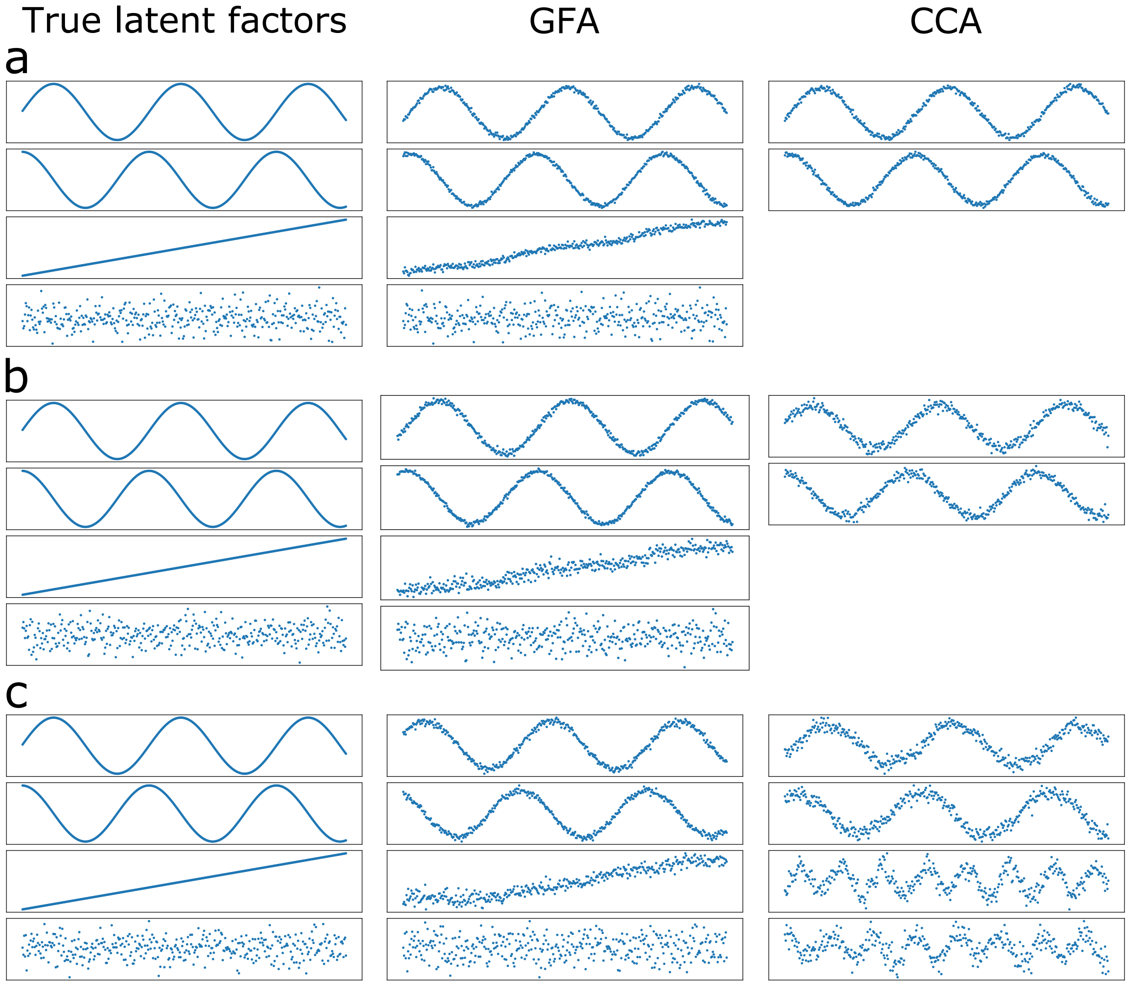

Fig. 4. True and inferred latent factors model obtained using CCA and GFA. **(Left column)** latent factors used to generate the data. **(Middle column)** latent factors inferred using the proposed GFA approach. **(Right column)** latent factors inferred using CCA. **(a)** experiment using complete data; **(b)** experiments using incomplete data, where 20% of the elements of $\mathbf{X}^{(1)}$ and 40% of the elements of $\mathbf{X}^{(2)}$ were randomly removed; **(c)** experiment using incomplete data, where the elements of $\mathbf{X}^{(1)}$ and $\mathbf{X}^{(2)}$ deviating more than 1σ (i.e. standard deviation) from the mean were removed.

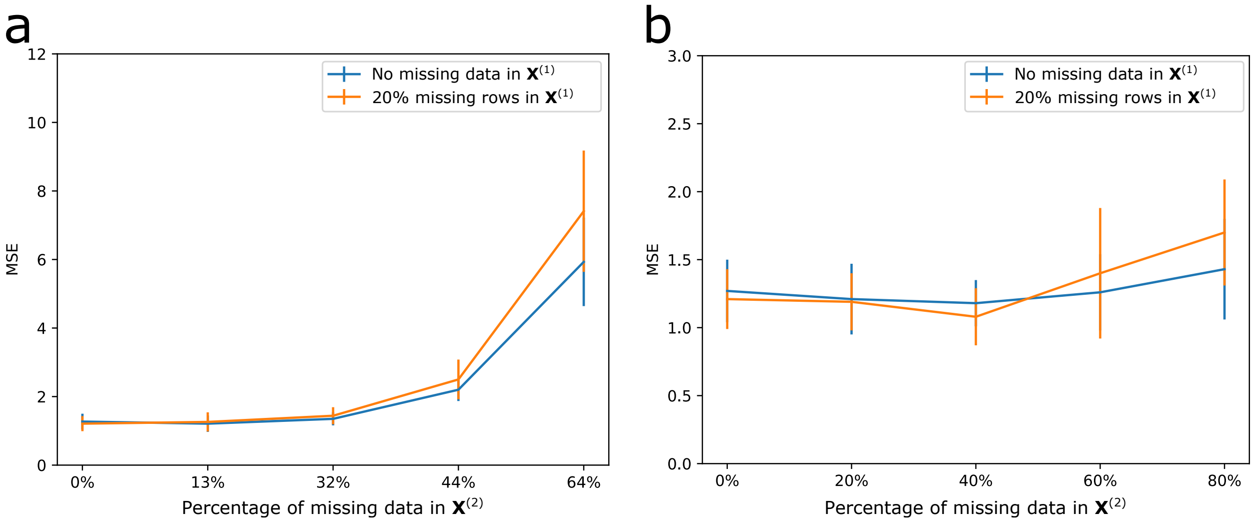

Fig. 5. Prediction errors of the proposed GFA approach on synthetic data with different percentages of missing data in $\mathbf{X}^{(2)}$. The performance of the model was measured by mean squared error (MSE), which was calculated between the true and predicted values of $\mathbf{X}^{(1)}$ on the test set. **(a)** The values of $\mathbf{X}^{(2)}$ were excluded from the tails of the distribution; **(b)** the values of $\mathbf{X}^{(2)}$ were randomly excluded. The blue (orange) line plot represents the averaged MSE obtained when there was no missing data (20% of rows were missing) in $\mathbf{X}^{(1)}$. The error bar represents the standard deviation of the MSEs obtained across 10 different random initialisations.

2.3. GFA experiments on the HCP data

Table 2: Most relevant shared and modality-specific factors obtained when HCP data was missing randomly (20% of the elements of the non-imaging matrix missing). Factors explaining more than 7.5% variance within any data modality were considered most relevant. A factor was considered shared if $0.001 \leq r_k \leq 300$, non-imaging (NI) specific if $r_k > 300$ or brain-specific if $r_k < 0.001$. rvar - relative variance explained; var - variance explained; r_k - ratio between the variance explained by the non-imaging and brain loadings in factor k.

Factors		**rvar** (%)		**var** (%)		r_k
		Brain	NI	Brain	NI	$\text{var}_{\text{NI}}/\text{var}_{\text{brain}}$
**Shared**	a	0.159	9.44	0.012	0.028	2.42
	b	0.065	18.152	0.005	0.005	11.32
	c	0.036	10.539	0.003	0.031	12.04
	d	0.015	39.330	0.001	0.117	105.10
**Brain**	a	13.531	6.60×10^{-5}	0.988	1.97×10^{-7}	1.99×10^{-7}
	b	12.269	0.001	0.896	4.19×10^{-6}	4.68×10^{-6}

Table 3: Most relevant shared and modality-specific factors obtained when brain connectivity data from the HCP dataset was missing for some subjects (20% of the subjects missing in the brain connectivity matrix). Factors explaining more than 7.5% variance within any data modality were considered most relevant. A factor was considered shared if $0.001 \leq r_k \leq 300$, non-imaging (NI) specific if $r_k > 300$ or brain-specific if $r_k < 0.001$. rvar - relative variance explained; var - variance explained; r_k - ratio between the variance explained by the non-imaging and brain loadings in factor k.

		**rvar** (%)		**var** (%)		r_k
	Factors	Brain	NI	Brain	NI	$\text{var}_{\text{NI}}/\text{var}_{\text{brain}}$
**Shared**	a	0.149	7.643	0.007	0.028	3.83
	b	0.034	16.550	0.002	0.060	36.19
	c	0.016	8.670	7.82×10^{-4}	0.031	40.11
	d	0.019	31.255	8.99×10^{-4}	0.113	125.82
**Brain**	a	15.625	0.0350	0.758	1.27×10^{-4}	1.67×10^{-4}
	b	14.979	0.177	0.727	6.42×10^{-4}	8.83×10^{-4}

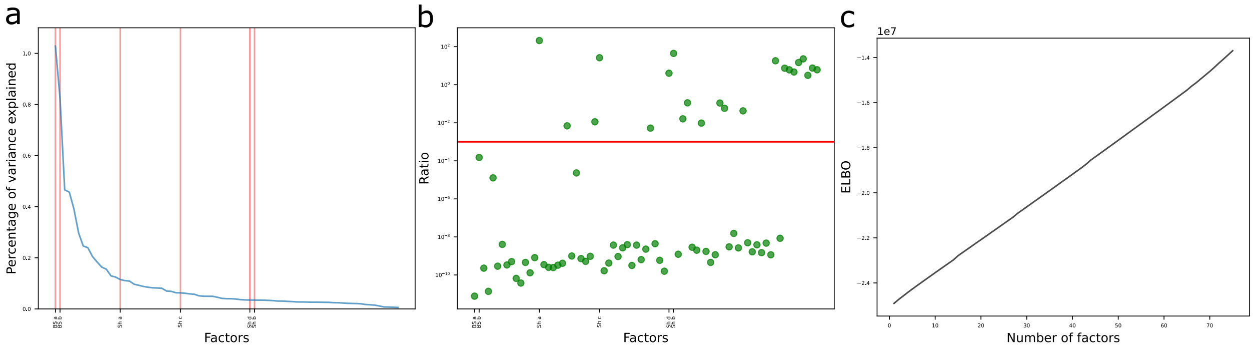

Fig. 6. Evidence supporting the criteria used to select the most relevant factors in the complete HCP data. **(a)** Percentage of variance explained by each factor, **(b)** ratio between the variance explained by the non-imaging and brain loadings of each factor (r_k) obtained in the complete data experiment of the main text , and **(c)** evidence lower bound (ELBO) when increasing the number of factors included in the model. The vertical lines in **(a)** were drawn for the factors considered most relevant (Fig. 6 and 7 of the main text). The horizontal line in **(b)** corresponds to ratio $r_k = 0.001$, the values below the line correspond to brain-specific factors and above to shared factors (no non-imaging specific factors were identified). BS - brain-specific; Sh - shared.

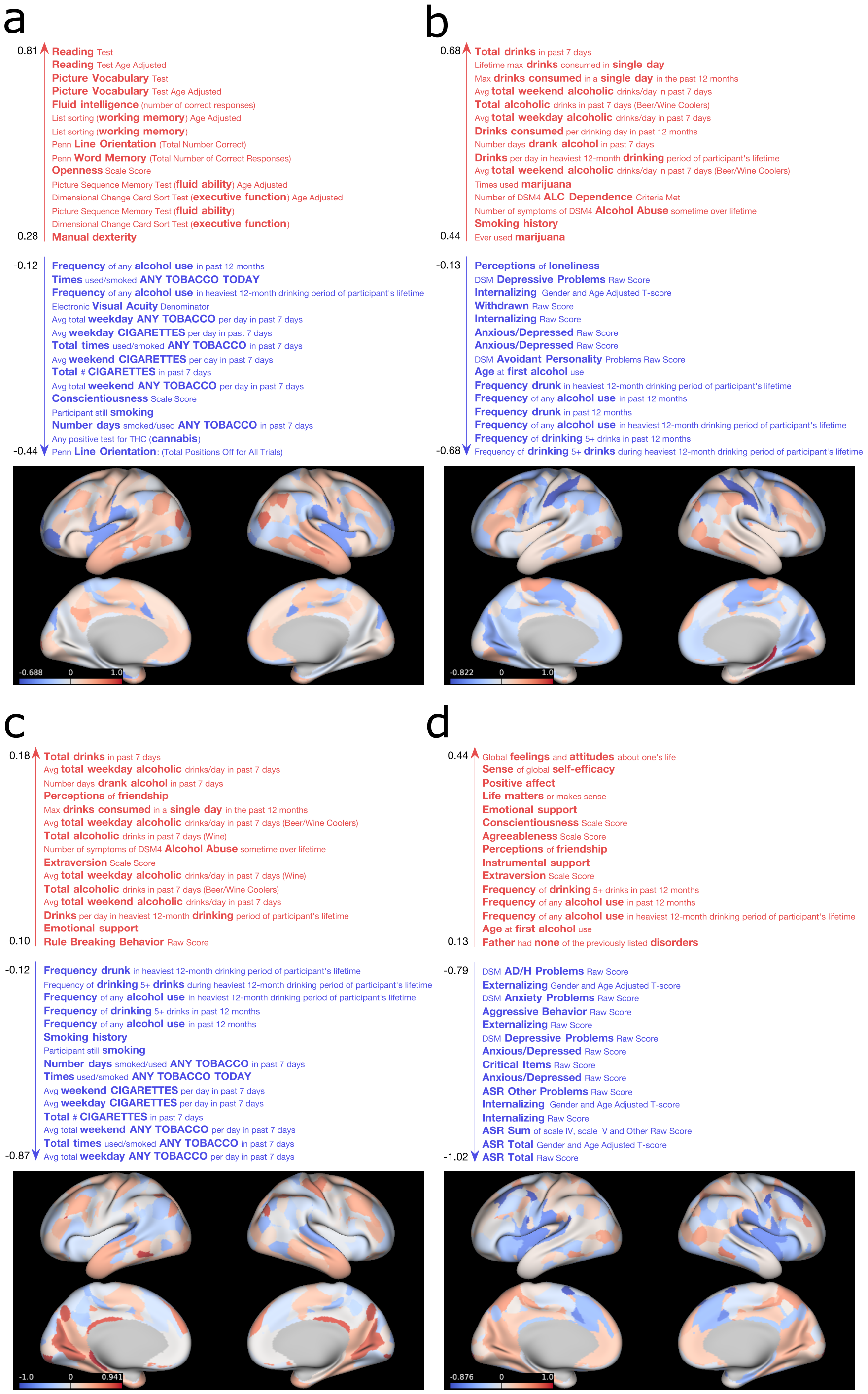

Fig. 7. Non-imaging measures and brain networks described by the first **(a)**, second **(b)**, third **(c)** and fourth **(d)** shared GFA factors obtained when HCP data was missing randomly (20% of the elements of the non-imaging matrix missing). For illustrative purposes, the top and bottom 15 non-imaging measures for each factor are shown. The brain surface plots represent maps of brain connection strength increases/decreases, which were obtained as described in supplementary Section 1.4.

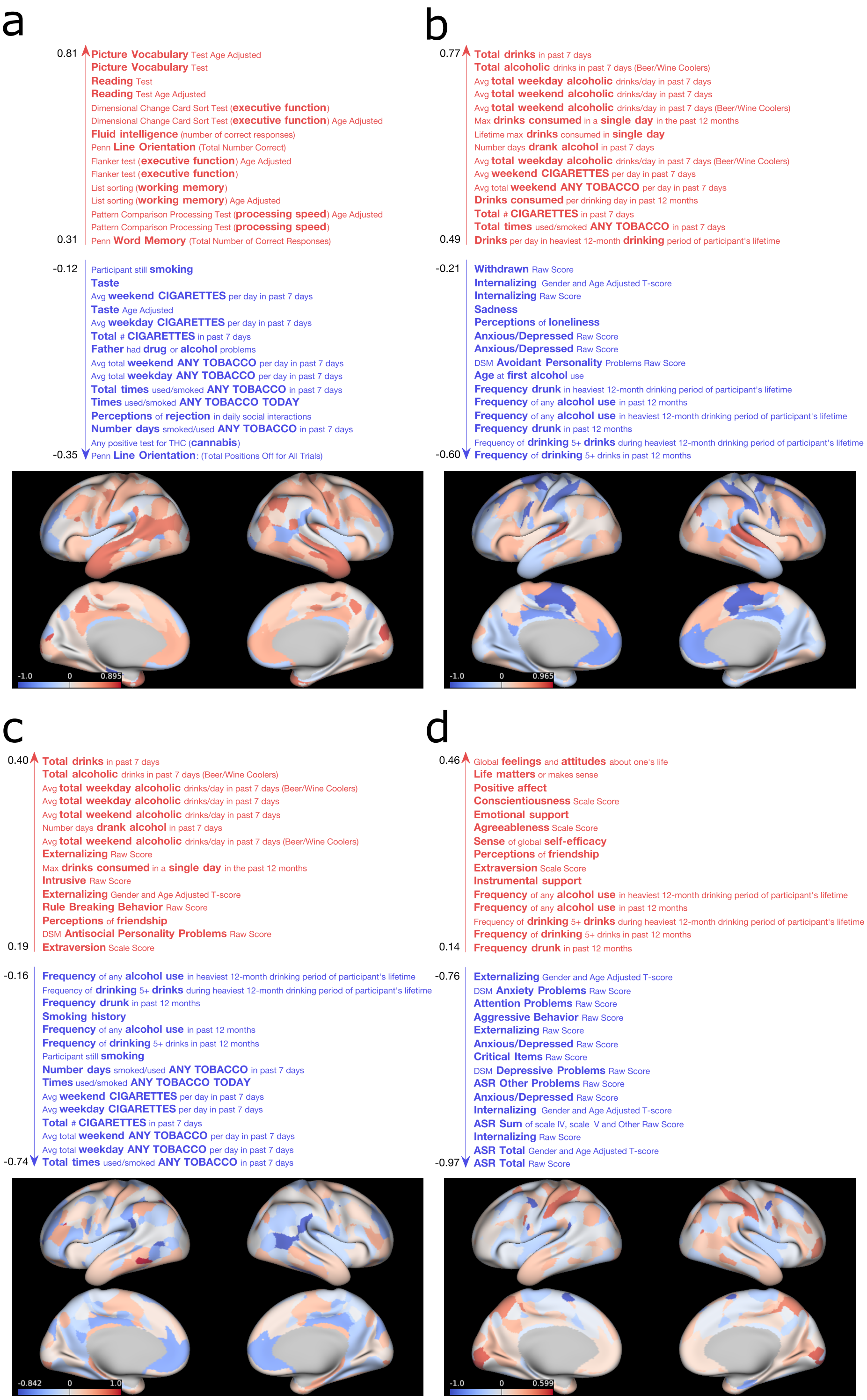

Fig. 8. Non-imaging measures and brain networks described by the first **(a)**, second **(b)**, third **(c)** and fourth **(d)** shared GFA factors obtained when brain connectivity data from the HCP dataset was missing for some subjects (20% of the subjects missing in the brain connectivity matrix). The top and bottom 15 non-imaging measures for each factor are shown. The brain surface plots represent maps of brain connection strength increases/decreases, which were obtained as described in supplementary Section 1.4.

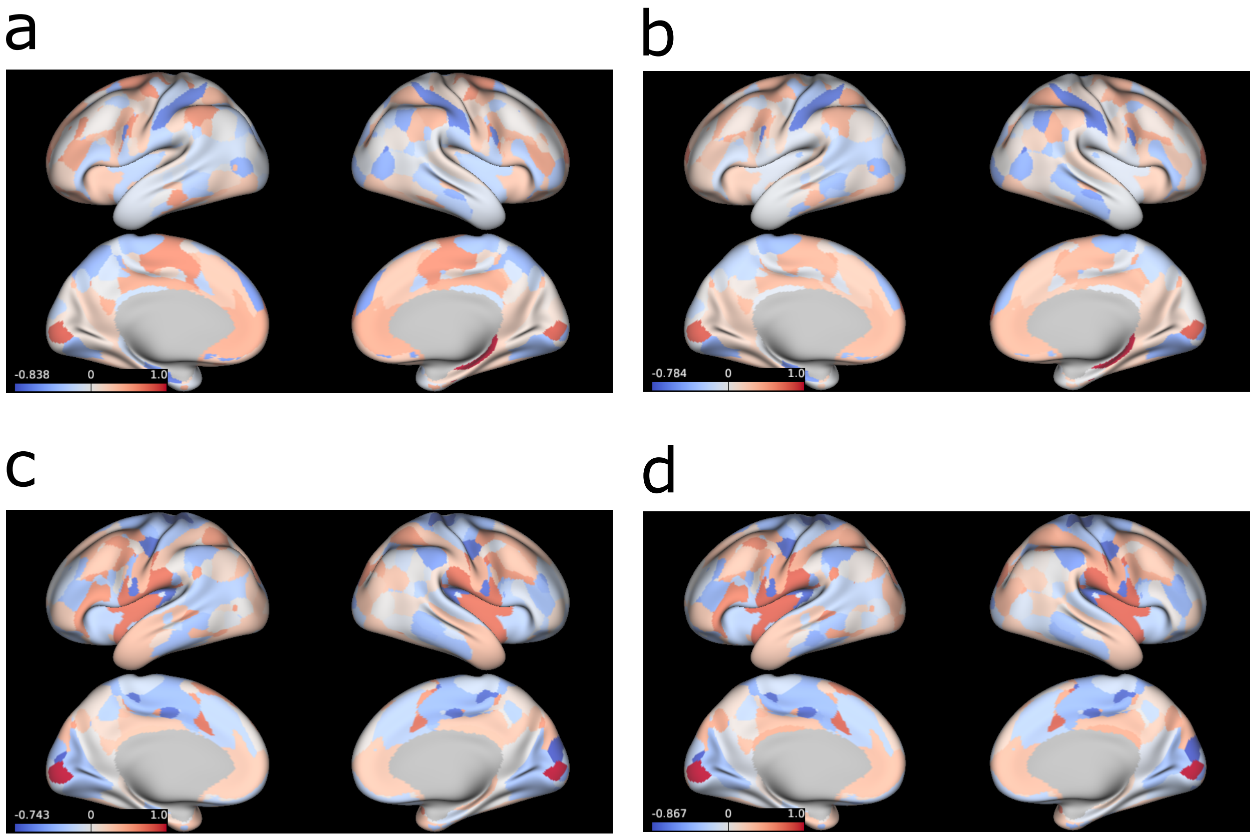

Fig. 9. Brain networks associated with the brain-specific GFA factors obtained when **(a,c)** HCP data was missing randomly (20% of the elements of the non-imaging matrix missing) or **(b,d)** brain connectivity data from the HCP dataset was missing for some subjects (20% of the subjects missing in the brain connectivity matrix). The brain surface plots represent maps of brain connection strength increases/decreases, which were obtained as described in supplementary Section 1.4.

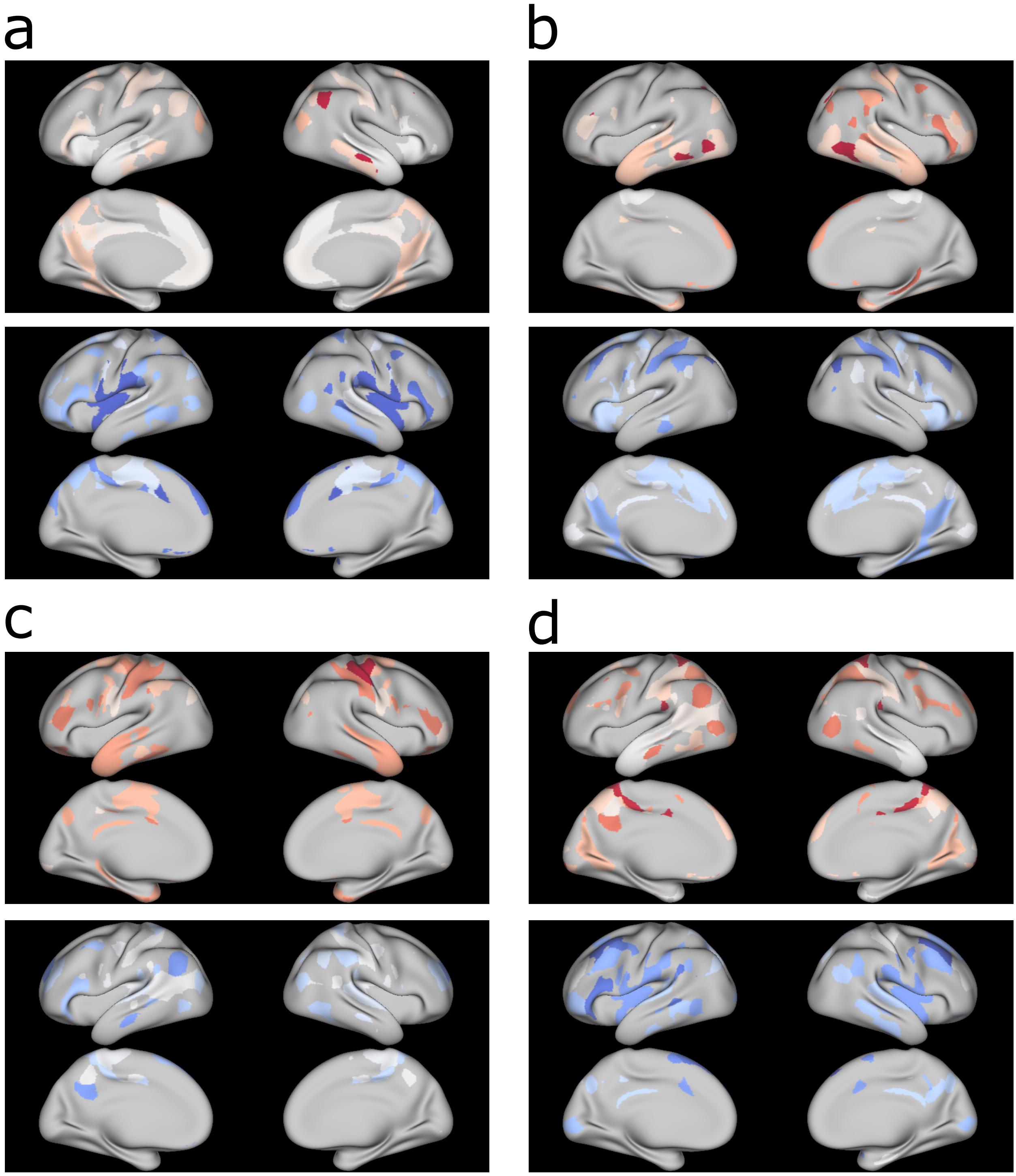

Fig. 10. Brain surface maps of the brain connection strength increases (red) and decreases (blue) of the first **(a)**, second **(b)**, third **(c)** and fourth **(d)** shared GFA factors obtained in the HCP experiment with complete data. The distribution of the brain connection strengths was thresholded at the 80th (red) and 20th percentile (blue).

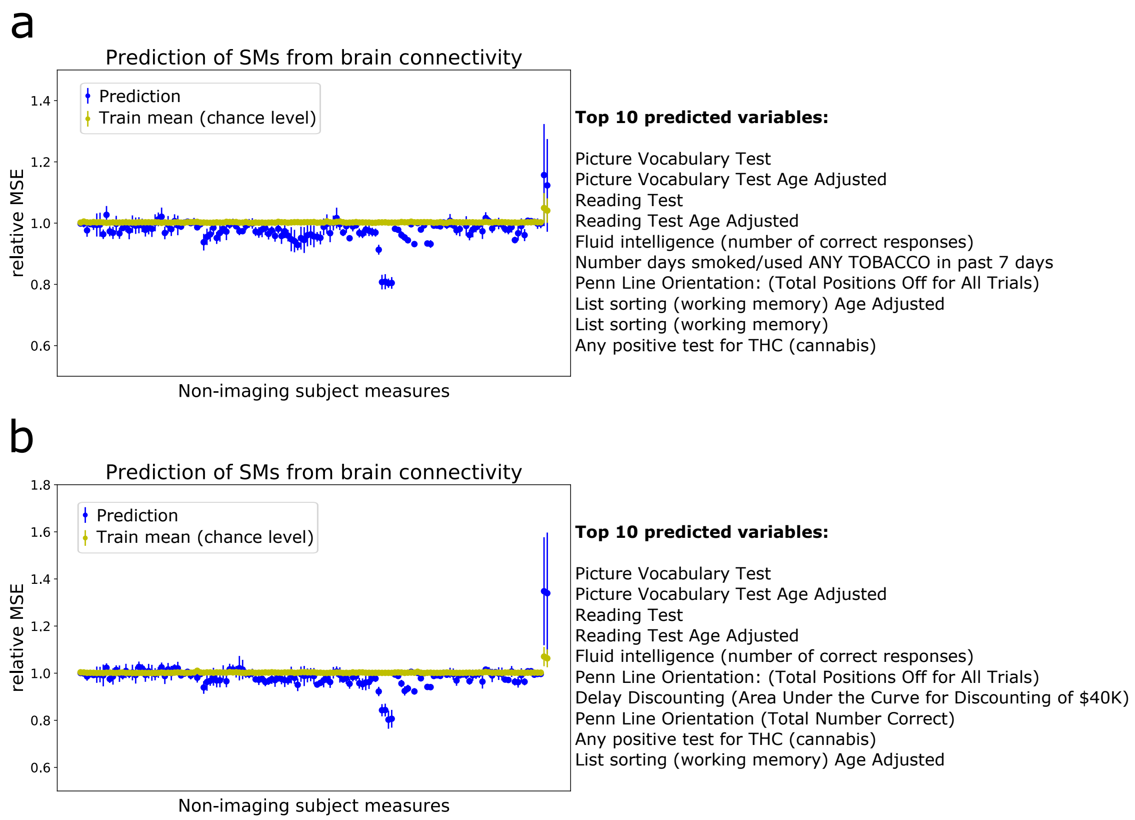

Fig. 11. Multi-output predictions of the non-imaging measures obtained when **(a)** HCP data was missing randomly (20% of the elements of the non-imaging matrix missing) or **(b)** brain connectivity data from the HCP dataset was missing for some subjects (20% of the subjects missing in the brain connectivity matrix). The top 10 predicted measures are described on the right. For each non-imaging measure, the mean and standard deviation of the relative MSE between the true and predicted values on the test set was calculated across different random initialisations of the experiments.

2.4. CCA experiments on the HCP data

To highlight the differences between GFA and CCA, we compared the CCA modes to the GFA factors obtained using complete data (Supplementary Fig. 12 and Supplementary Table 4). To interpret the association captured by each CCA mode, we correlated the non-imaging measures and brain connectivity variables with the canonical scores obtained for each data modality (as in Smith et al. (2015)), respectively. The non-imaging measures identified by the first, second and third CCA modes (Supplementary Fig. 12a,b,c) share similarities with the top and bottom non-imaging measures obtained in the first GFA factor (Fig. 6a of the main text). However, the positive brain loadings on the posterolateral and medial default mode networks in the first GFA factor are split between the first and third CCA modes, respectively. The fourth CCA mode is in many respects the inverse of the second GFA factor, both in terms of brain regions (default mode and insular areas) and non-imaging measures, which are dominated by alcohol use (with recent and lifetime use loading at opposite ends of each mode). The fifth CCA mode related most strongly inattention, aggression and antisocial behaviour to positive loadings on posterior insula,

and inferior, superior and medial frontal regions. The fourth GFA factor contained these non-imaging measures and low mood/internalising as well. Moreover, the brain loadings in lateral prefrontal and insular cortex were similar across the fifth CCA mode and the fourth GFA factor, as were attention problems and aggression, with conscientiousness loading in the opposite direction.

Finally, the first GFA factor (related to CCA modes 1-3) replicates the findings found by Smith et al. (2015) using CCA applied to approximately 500 subjects (first release of the HCP dataset). Both of these contained loadings related to cognitive performance and tobacco or cannabis use, and brain loadings on default mode areas. Some remaining non-imaging measures in Smith et al.'s factor appeared in our fourth GFA factor (related to life satisfaction and aggression), which strongly related to different forms of psychopathology.

Table 4: Pearson's correlations between the most relevant GFA factors (in the complete data experiment described in the main text) and the CCA modes (Supplementary Fig. 12) obtained by applying CCA as described in supplementary Section 1.3. The values in bold represent the highest absolute correlations between a given CCA mode and the GFA factors.

		**GFA**					
		**Shared**				**Brain-specific**	
		a	b	c	d	a	b
**CCA**	a	**0.605**	0.011	0.105	0.064	0.093	0.347
	b	**0.380**	0.112	0.050	0.190	0.093	0.081
	c	0.231	0.112	0.206	0.065	**0.299**	0.048
	d	0.009	**0.191**	0.039	0.061	0.083	0.036
	e	0.052	0.092	0.115	0.173	0.031	**0.386**

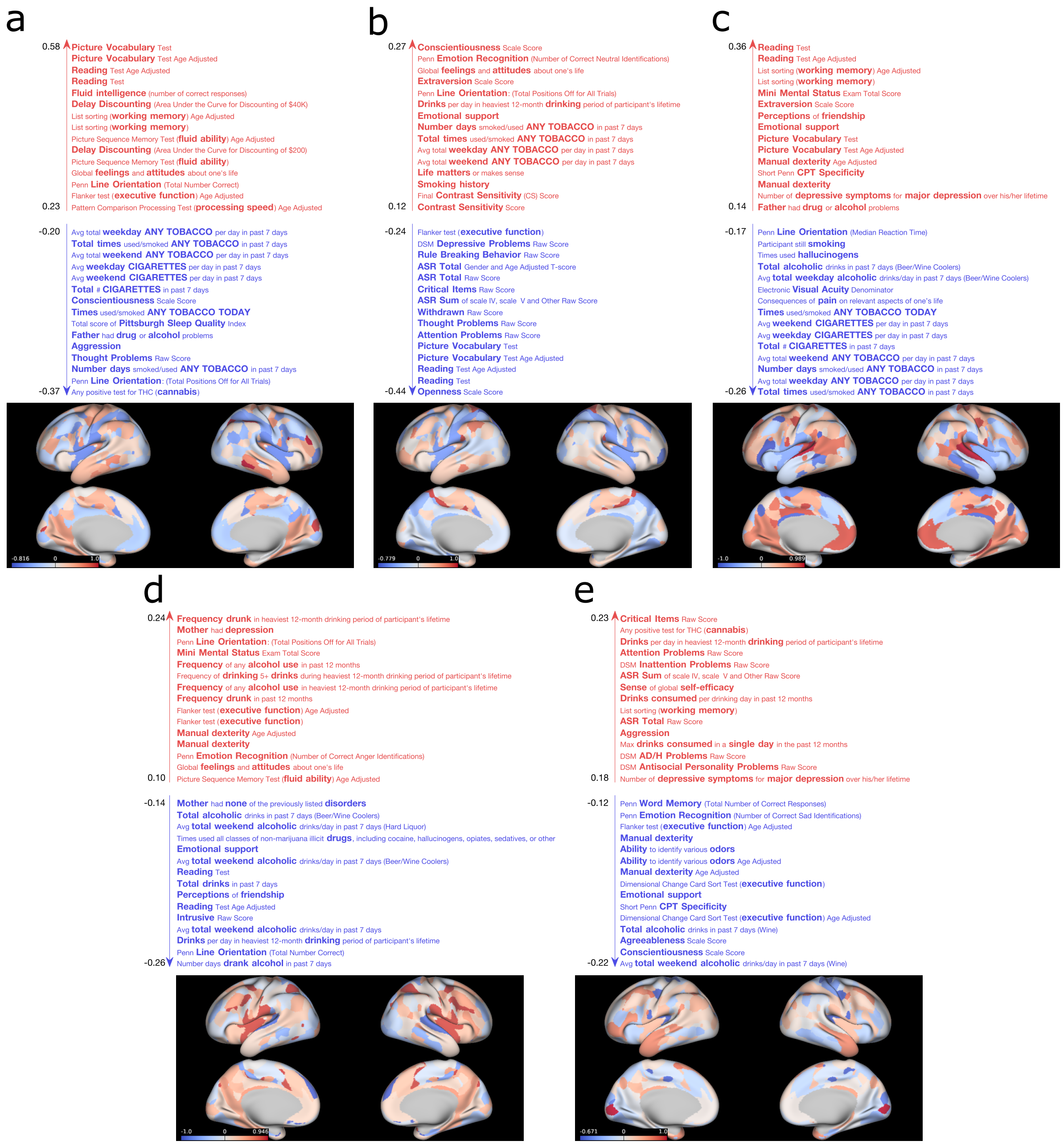

Fig. 12. Non-imaging measures and brain networks correlated with the CCA modes obtained by applying CCA as described in supplementary Section 1.3. For illustrative purposes, the top and bottom 15 non-imaging measures for each factor are shown. The brain surface plots represent maps of brain connection strength increases/decreases, which were obtained as described in supplementary Section 1.4.

2.5. Alcohol use loadings of the second GFA factor

The second GFA factor (Fig. 6b of the main text) has puzzlingly opposing loadings of frequency of alcohol use versus total alcohol drunk in the last seven days. This is probably because the distributions of "total amount" answers are very skewed, with most subjects reporting zero, hence a lot of variance can be explained by this rather paradoxical set of

loadings (Supplementary Fig. 13). Alternatively, it might be that these alcohol use items represent two different behaviours, where "total amount" answers are related to a more short-term alcohol use and the "frequency" answers might represent more long-term and consistent alcohol use.

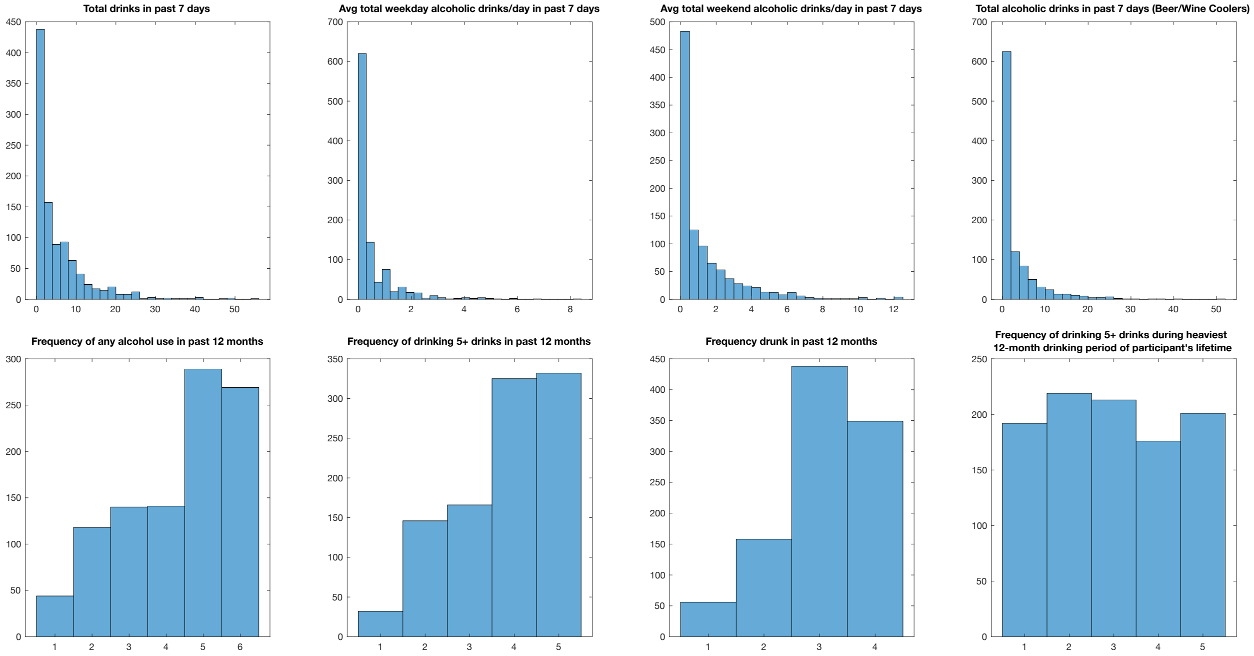

Fig. 13. Histograms of the top 4 variables **(top)** and bottom 4 variables **(bottom)** of the second shared GFA factor displayed in Fig. 6b of the main text.

References

Smith, S.M., Nichols, T.E., Vidaurre, D., Winkler, A.M., Behrens, T.E.J., Glasser, M.F., Ugurbil, K., Barch, D.M., Van Essen, D.C., Miller, K.L., 2015. A positive-negative mode of population covariation links brain connectivity, demographics and behavior. Nature Neuroscience 18, 1565–1567. doi:10.1038/nn.4125.

Winkler, A.M., Webster, M.A., Vidaurre, D., Nichols, T.E., Smith, S.M., 2015. Multi-level block permutation. NeuroImage 123, 253–268. doi:10.1016/j.neuroimage.2015.05.092.
